# Raising to conformity without strictness: is it achievable?

**DOI:** 10.3389/fpsyg.2025.1568132

**Published:** 2025-04-14

**Authors:** Marta Alcaide, Oscar F. Garcia, Olga Gomez-Ortiz, Fernando Garcia

**Affiliations:** ^1^Department of Methodology of the Behavioral Sciences, University of Valencia, Valencia, Spain; ^2^Department of Developmental and Educational Psychology, University of Valencia, Valencia, Spain; ^3^Department of Psychology, University of Cordoba, Cordoba, Spain

**Keywords:** parenting dimensions, child adjustment, adolescent children, adult children, conformity values, psychosocial adjustment

## Abstract

**Background:**

The prevailing belief that parental strictness is optimal for children is not uniformly supported by recent research. Contrary to the traditional notion that strictness is necessary to ensure children’s conformity to social norms, contemporary studies question its necessity. This study aims to analyze how two main parenting dimensions, warmth and strictness, are related to the psychosocial adjustment of children.

**Method:**

A sample of 1,224 Spanish adolescents and young adults was examined, divided into two groups: adolescents aged 12–18 years (51.14%) and young adults aged 19–35 years (48.86%). Adolescent participants were recruited from high schools while young adults were recruited from university courses. Children (i.e., adolescent and young adult offspring) responded to an online questionnaire that included all measures: parenting dimensions (warmth and strictness) and children’s psychosocial adjustment criteria (emotional self-concept, self-esteem, social competence, and conformity). Power analyses (*a priori* and sensitivity analyses) were applied to ensure sufficient sample sizes to achieve adequate power. Cohen’s *d* values from correlation analyses and multiple linear regression analyses were performed. *Beta* confidence intervals were analyzed to relate parenting dimensions, sex and age to self-concept, self-esteem, social competence, and conformity.

**Results:**

The statistical analysis plainly indicated that parental warmth was positively associated with criteria for child psychosocial adjustment, including self-esteem, social competence, and conformity. This relationship was consistent across both adolescents and young adults. Conversely, parental strictness was either negatively related to or not significantly associated with these criteria.

**Conclusion:**

This study clearly suggests that, completely contrary to expectations that strict parenting might be a need component to achieving psychosocial adjustment, parental warmth, rather than strictness, is more effective in promoting children’s psychosocial adjustment.

## 1 Introduction

Parenting literature has continuously recognized the importance of socialization for individuals (Pinquart and Kauser, 2018; Baumrind, 1978; Freud, 1933). During childhood, the primary influence comes from the family environment. As children transition into adolescence, peer influence significantly increases, and in adulthood, relationships with parents shift from being defined by legal requirements (with parents acting as guardians) to becoming more informal (Steinberg, 2001; Darling and Steinberg, 1993; Maccoby, 1992; Baumrind, 1991a; Lamborn et al., 1991). Rather than being based on parental authority and child obedience, intergenerational relationships during the adult years and even into later life are grounded in parental and filial maturity characterized by aspects such as contact, closeness, and assistance—though they can sometimes be marked by conflict (Padilla-Walker et al., 2014; Padilla-Walker et al., 2012; Aquilino, 2006; Zarit et al., 2005; Belsky et al., 2001). Research has examined the relationship between parental socialization and child functioning, particularly in relation to conformity to social norms and other aspects of adjustment. Overall, the significance of the family has been identified as crucial acting as either a protective or risk factor (Steinberg, 2005; Steinberg and Morris, 2001; Darling and Steinberg, 1993). Parental strictness and warmth are conceptualized as key variables that explain the differences observed in terms of conformity and other criteria of psychosocial adjustment (Garcia and Gracia, 2009; Steinberg, 2005; Steinberg et al., 1994; Lamborn et al., 1991). Traditionally, parental strictness was identified as beneficial for preventing maladjustment in children (Clark et al., 2015; Dwairy and Achoui, 2006; Chao, 2001; Deater-Deckard et al., 1996; Baumrind, 1972). When combined with parental warmth, strictness has been associated with optimal levels of psychosocial adjustment (Lamborn et al., 1991; Eccles et al., 1991; Steinberg et al., 1989; Maccoby and Martin, 1983; Baumrind, 1978). Nevertheless, recent studies challenge the widely accepted view of the benefits of parental strictness, suggesting that parental warmth alone—without strictness—may also foster psychosocial adjustment in children and help prevent deviant behavior (Pinquart and Lauk, 2024; Villarejo et al., 2024; Garcia et al., 2020b; Lund and Scheffels, 2019; Pinquart and Kauser, 2018; Calafat et al., 2014).

Parents try to transmit social norms and values to their children, aiming to prepare them to become competent adults in the society in which they live (Rudy and Grusec, 2001; Bugental and Goodnow, 1998; Darling and Steinberg, 1993; Maccoby, 1992; Baumrind, 1978). The internalization of social norms is the process by which individuals prioritize conformity to behavioral standards and adjust their actions to align with social standards (Etzioni, 2000; McAdams, 1997). Conformity values, characterized as universal social values, involve restraining actions, tendencies, and impulses that could disrupt or harm others and violate social expectations (Schwartz, 2010; Schwartz, 2007; Schwartz, 2006). Conformity values include respect, self-discipline, politeness, and honoring parents and older adults (Katz-Gerro et al., 2017). By the internalization of social norms, the individuals also become aware of the effects on others when they do not behave in accordance with the norm (Etzioni, 2000; McAdams, 1997). Conformity to social norms can lead to social approval, an external reward that is positively related to happiness (Stavrova et al., 2013; Cialdini and Goldstein, 2004) and behaviors related to helping others (Oarga et al., 2015). Furthermore, conformity to social norms is associated with good adjustment (Oarga et al., 2015; Christensen et al., 2004; Cialdini et al., 1990).

### 1.1 Evidence based on the two-dimensional parenting framework to assure conformity

The two-dimensional framework identifies two theoretically orthogonal, or unrelated, dimensions: warmth and strictness (Maccoby and Martin, 1983). Parental warmth, often referred to as responsiveness (Baumrind, 1982), supportiveness (Garcia and Gracia, 2014), acceptance (Symonds, 1939), or love (Schaefer, 1959), involves showing approval and support to children while using dialog and reasoning to establish rules and limits (Martinez-Escudero et al., 2023; Martinez et al., 2017; Baumrind, 1965). In contrast, parental strictness—also known as control (Schaefer, 1959), firm control (Steinberg, 2005), inflexibility (Sears et al., 1957), or hostility (Baldwin, 1955)—refers to the extent to which parents implement discipline, exert control, supervise behavior, enforce rules, and maintain authority over their children (Harma et al., 2025; Reyes et al., 2023; Baumrind, 1965). Parents can be described in terms of both warmth and strictness. Authoritative parents are characterized as both strict and warm (Darling and Steinberg, 1993; Maccoby and Martin, 1983). In contrast, authoritarian parents are strict but lack of warmth (Pinquart and Kauser, 2018; Chao, 2001). Neglectful parents are neither strict nor warm (Lamborn et al., 1991; Maccoby and Martin, 1983), while indulgent parents are not strict, but warm (Palacios et al., 2022; Martinez et al., 2021). Furthermore, the two-dimensional framework of theoretical parenting model also helps researchers to define different individual parenting practices based on two main parental axes (Martinez-Escudero et al., 2020; Darling and Steinberg, 1993; Maccoby and Martin, 1983; Baumrind, 1965). For example, parenting practices named as psychological control are characterized by strictness and lack of warmth, which are related to authoritarian parents (Barber, 1996; Schaefer, 1959). Parenting practices of behavioral control are characterized by high strictness and high warmth, which are related to authoritative parents (Steinberg, 2005; Silk et al., 2003). A distinctive aspect of behavioral control compared to psychological control has been identified as parenting monitoring explained by children’s spontaneous disclosure of information to their parents (authoritative parenting), but not by parents’ tracking and scrutiny efforts (authoritarian parenting) (Stattin and Kerr, 2000; Kerr et al., 1999). Parenting practices such as reasoning and dialog to limit the child’s incorrect behaviors are positively related to the parental warmth dimension, which is related to authoritative and indulgent parents (Garcia and Gracia, 2009; Grusec and Lytton, 1988).

Research has identified that variations in the degree to which adolescents internalize values and behave in socially desirable ways can be consistently related to parental socialization (Li et al., 2023; Martinez and Garcia, 2007; Knafo, 2003; Grusec and Goodnow, 1994). Research conducted primarily in the United States with European-American families has identified that strictness can help deter the development of deviance, problems, and maladjustment, but only its combination with parental warmth (i.e., authoritative parenting) is associated with the greatest benefits in different components of psychosocial adjustment (Lamborn et al., 1991; Eccles et al., 1991; Steinberg et al., 1989). The authoritative style has been related to high self-esteem (Barber et al., 1992) and psychosocial maturity, including a strong sense of self-reliance, work orientation, and social competence (Lamborn et al., 1991; Steinberg et al., 1989). Evidence from studies of European-American families also revealed that adolescents raised by authoritarian parents benefit from strictness—similar to that seen in authoritative families—and tend to report high obedience and conformity to social standards, with lower rates of school misconduct, drug use, and a positive orientation toward school. It is argued that the low levels of social maladjustment reported by adolescents with authoritative and, to a similar extent, authoritarian parents, may be due to the component of strictness, which acts as a protective factor against externalized problems (Lamborn et al., 1991; Patterson and Stouthamerloeber, 1984). Nevertheless, European-American adolescents with authoritarian parents tend to report more personal problems, such as poor self-conceptions and somatic stress, probably due to the lack of parental warmth that distinguishes authoritarian parenting from authoritative parenting (Darling and Steinberg, 1993; Lamborn et al., 1991).

### 1.2 Cultural differences in the optimal parenting

Nevertheless, parenting characterized by strictness and warmth (i.e., authoritative parenting) is not always associated with optimal child adjustment across all cultural contexts (Pinquart and Lauk, 2024; Palacios et al., 2022; Garcia et al., 2019; Chao, 2001; Darling and Steinberg, 1993). According to some cross-cultural research, the use of parental strictness, even without warmth (i.e., authoritarian parenting), is positively associated with child adjustment. For instance, studies conducted the United States with ethnic minority groups, such as Asian-American (Chao, 2001; Chao, 1994) and African-American families (Deater-Deckard et al., 1996; Baumrind, 1972), as well as evidence from Arab societies (Dwairy and Achoui, 2006; Dwairy et al., 2006), show that strictness without warmth is related to adequate child adjustment in terms of academic achievement (Chao, 2001; Steinberg et al., 1994), social behavior, interpersonal relationships (Dwairy et al., 2006), assertiveness and independence (Baumrind, 1972).

Additionally, recent evidence about parenting in European and Latin American countries has found that parental warmth without strictness is associated with optimal personal and social adjustment in children (Martin-Blesa et al., 2024; Garcia et al., 2020a; Martinez et al., 2020; Calafat et al., 2014; Lila et al., 2007). Adolescents from warm but not strict parents (i.e., the indulgent style) have the same or better adjustment than their peers with parents characterized by warmth accompanied by strictness (i.e., the authoritative style) in different indicators of psychosocial adjustment. The indulgent style has been related to low levels of aggression (Alcaide et al., 2023), hostile sexism (Alcaide et al., 2023; Gimenez-Serrano et al., 2022), hostility (Garcia and Serra, 2019) emotional unresponsiveness (Reyes et al., 2023), and delinquency (Climent-Galarza et al., 2022), and high levels of social competence (Villarejo et al., 2024), and social self-concept (Fuentes et al., 2022). High levels of strictness—which distinguish authoritative from indulgent parenting—may be unnecessary and even detrimental, whereas the use of warmth is associated with important benefits for child adjustment (Gallarin et al., 2021; Garcia et al., 2019; Pinquart and Kauser, 2018).

Other benefits related to the indulgent parenting style have also been identified in recent research mainly with European and Latin American families (Pinquart and Lauk, 2024; Garcia et al., 2020b; Lund and Scheffels, 2019; Calafat et al., 2014; Garcia and Gracia, 2009). Children from warm but not strict families (indulgent parenting) have advantages compared to their counterparts who also come from warm homes but combine warmth with strictness (authoritative parenting) in areas such as emotional self-concept concept (Martin-Blesa et al., 2024), family self-concept (Perez-Gramaje et al., 2020), and academic-professional self-concept (Martinez-Escudero et al., 2023). Additionally, they report low levels of negative self-esteem (Reyes et al., 2023) and negative self-efficacy (Palacios et al., 2022). Despite research with European-American families predicting that low strictness would be a risk factor for child adjustment, these emerging results indicated that negative scores on psychosocial adjustment were associated not with a lack of parental strictness related to indulgent parenting, but rather with both parenting styles that had a lack of warmth—authoritarian and neglectful parenting (Garcia et al., 2024; Fuentes et al., 2022; Palacios et al., 2022).

### 1.3 Parenting and their possible long-term effects

Despite parental socialization is over when adolescents reach the adult age, not all of them have internalized the social values and behave according to social norms (Climent-Galarza et al., 2022; Maccoby, 1992; Baumrind, 1978). For example, some research indicates that levels of drug use may not decrease after adolescence; instead, they may remain constant or even increase into young adulthood (Peacock et al., 2018). As adult members of society, adult children, without parental supervision and care, face different challenges, such as professional development and starting a family (Mañez et al., 2024; Buchinger et al., 2022). Nevertheless, parental socialization can have a long-term impact on adult children for several reasons (Krauss and Orth, 2024; Maccoby, 1992; Freud, 1933).

The years from birth through childhood and adolescence are critical for parental socialization, during which enduring social skills, personality traits, orientations, and values are developed (Krauss and Orth, 2024; Darling and Steinberg, 1993; Maccoby, 1992). Although children influence their parents, the power asymmetry between adults and children is enormous (Gómez-Ortiz et al., 2025; Nomaguchi and Milkie, 2020). Parents inevitably have a decisive impact on their children, particularly since children do not initiate their own actions to the same extent as their parents do or should do (Steinberg, 2001; Baumrind, 1978). This influence is especially salient in childhood due to the high level of plasticity during this developmental time. However, parents remain crucial during adolescence, a period when adolescents are increasingly influenced by their peers, who help them in their search for autonomy and personal identity (Riquelme et al., 2018; Erikson, 1968). Adolescence is characterized by significant biological and social changes, during which adolescents compare themselves to slightly older peers and recognize differences (Steinberg and Morris, 2001). Some degree of psychosocial vulnerability is often associated with adolescence, including declines in self-concept and self-esteem (Esnaola et al., 2020; Orth et al., 2015), behavioral problems (Tao et al., 2021), low engagement in school (Veiga et al., 2023), or drug use (Calafat et al., 2014).

Despite the significant influence of peers on adolescents—which can have important benefits but may also lead to problems when adolescents adopt deviant standards from their peer group—parental socialization is considered an important protective factor against adolescent vulnerability (Riquelme et al., 2018; Baumrind, 1991b). Conformity values reflect one’s orientation toward social standards without causing harm or annoyance to others, acting as a protective factor against vulnerability. Research based on European-American families indicates that parental strictness favors conformity to social norms and offers protection against problems, while parental warmth fosters child individuality and personal well-being (Steinberg et al., 1994; Lamborn et al., 1991). However, current studies suggest that protection against psychosocial vulnerabilities (e.g., alcohol and drug use) is associated with warmth, even in the absence of strictness (Garcia et al., 2020b; Calafat et al., 2015). Partly due to the malleability of childhood as well as the protective or risk role against psychosocial vulnerability of adolescence, parents, as socializing agents, might a unique role—positive or negative—to help their children become socially competent and well-adjusted adults (Garcia et al., 2024; Darling and Steinberg, 1993; Maccoby, 1992).

### 1.4 The present study

Despite developmental theories suggesting that early experiences have long-term effects on adult development, few studies have focused on the consequences of parenting beyond adolescence (Garcia et al., 2018; Stafford et al., 2015). Although the age range defining adolescence has been debated and includes new conceptualizations that even extend it to age 24 (Sawyer et al., 2018), socialization literature recognizes that reaching the adult age represents a fundamental shift in an individual’s societal obligations (Climent-Galarza et al., 2022; Bugental and Goodnow, 1998; Darling and Steinberg, 1993; Maccoby, 1992; Baumrind, 1978). This includes changes in interactions with teachers, which differ between university and high school (Steinberg, 2024), as well as with family, as parental socialization typically concludes, although parent–child relationships often continue into adulthood and later years (Zarit et al., 2005). Therefore, an important point to consider is whether the relationship between upbringing during parental socialization and current adjustment in adulthood follows a similar or different pattern compared to what is observed during adolescence. The evidence about parenting and its consequences on adult children is limited, particularly in studies that examine both adolescents and young adults simultaneously (Stafford et al., 2015; Hamon and Schrodt, 2012). Another important issue is whether, as classic literature suggests, a combination of parental strictness and warmth is beneficial for children in achieving conformity to social norms and scoring positively on other criteria of psychosocial adjustment (Darling and Steinberg, 1993; Lamborn et al., 1991). Recent studies, primarily from European and Latin American countries, call into question the benefits of parental strictness; instead, they propose that parental warmth may be sufficient to encourage children to favor optimal conformity to social norms and develop good adaptation (Garcia et al., 2019; Calafat et al., 2014). The present study aimed to examine the relationship between parenting dimensions (i.e., warmth and strictness) and child psychosocial adjustment (i.e., emotional self-concept, self-esteem, social competence, and conformity) during adolescence and young adulthood. Based on recent evidence, it is expected that: (i) parental warmth will be positively associated with children’s psychosocial adjustment (i.e., higher emotional self-concept, self-esteem, social competence, and conformity); and (ii) parental strictness will not be significantly related to children’s psychosocial adjustment.

## 2 Method

### 2.1 Participants and procedure

The sample was composed of 1,224 participants from Spain, including 723 women (59.10%) and 501 men (40.90%). Participants were adolescent and adult children ranging from 12 to 35 years (*M* = 20.03, *SD* = 4.54), divided into two age groups: 626 adolescents aged 12 to 18 years (*M* = 16.84, *SD* = 1.59), of which 372 were women (59.42%); and 598 young adults aged 19 to 35 years (*M* = 23.61, *SD* = 3.77), of which 351 were women (58.70%). Age-specific and sex-specific descriptives were as follows: for adolescent women, *M* = 16.84, *SD* = 1.59; for adolescent men, *M* = 16.28, *SD* = 1.75; for young adult women, *M* = 23.25, *SD* = 3.59; and for young adult men, *M* = 24.13, *SD* = 3.97 (see Table 1). An *a priori* power analysis was conducted (Garcia et al., 2020a; Garcia et al., 2008; Erdfelder et al., 1996) to determine the sample size necessary to detect a medium-small effect size for Pearson correlation analysis (*R*^2^ = 0.028, *R* = 0.1676) with a statistical power of 0.95 (1 − β = 0.95) and with conventional values for Type I and Type II statistical inference errors (α = 0.05; 1 − β = 0.95). The results of the a priori power analysis showed that a minimum sample of 452 participants was necessary (Faul et al., 2009; Garcia et al., 2008; Pérez et al., 1999; Lamborn et al., 1991; Cohen, 1977). The sample size of the present study (i.e., *N* = 1,224) exceeds the minimum sample size required.

**Table 1:** Participants characteristics for age, sex and frequency.

	Adolescents		Young adults	
	Women	Men	Women	Men
*N*	372	254	351	247
Percentage	59.42	40.58	58.70	41.30
*M* years	16.84	16.28	23.25	24.13
*SD* years	1.59	1.75	3.59	3.97

A sensitivity power analysis showed that for the total sample of this study (*N* = 1,224), with a statistical power of 0.95 (1 − β = 0.95) and setting the conventional values for Type I and Type II statistical inference errors (α = 0.05; 1 − β = 0.95), it is possible to detect a nearly small effect size (*R*^2^ = 0.0105, *R* = 0.1026) for Pearson correlation analysis (Fuentes et al., 2022; Garcia and Gracia, 2010; Faul et al., 2009). Additionally, a sensitivity power analysis for multiple linear regression showed that, by establishing the conventional values for Type I and Type II statistical inference errors (α = 0.05; 1 − β = 0.95) and considering four independent variables with 1,219 degrees of freedom for error, it is also possible to detect a nearly small effect size (*R^2^* = 0.0150, *R* = 0.1224). G*Power 3.1 software was used to calculate the statistical power (Faul et al., 2009).

Adolescents were recruited from high schools. Specifically, a random selection was conducted based on a list of high schools from the largest metropolitan area in Spain. The heads of the selected high schools were contacted to invite them to participate in the study (only one refused to participate). If a head refused to participate in the research, another high school from the complete list was chosen until the required sample size was achieved (Garcia et al., 2024; Martinez et al., 2021). Young adults were recruited from university courses (Koutra et al., 2022; Manzeske and Stright, 2009) and received course credit for participating. Data were gathered using an online survey with mandatory responses that was hosted on the university website during the 2021–2022 and 2022–2023 academic years. The online survey was administered during class time. Participants took about 45–50 min to complete the online survey. The questionnaires were assessed for uncertain answer patterns, such as implausible inconsistencies between negatively and positively formulated answers (Garcia et al., 2011; Tomás and Oliver, 2004).

All participants signed a declaration of agreement, and parental consent was also required for adolescent participants. The research adhered to the principles of the Helsinki Declaration. All participants in the present study met the following requirements: (a) they were Spanish, as were their parents and grandparents; (b) they lived in two-parent nuclear families with a mother or principal female caregiver and a father or principal male caregiver; and (c) they completed the same questionnaires. Participants were informed that participation was voluntary and that they could withdraw from the study at any time if they chose to do so (Climent-Galarza et al., 2022; Garcia et al., 2021). Anonymity and confidentiality were guaranteed for all respondents. To safeguard data protection measures, identifiers and survey data were kept in separate files, access to directories was secured with a secret code, and sensitive archives were encrypted.

### 2.2 Measures

#### 2.2.1 Parental socialization

Parental socialization was assessed through parental warmth and parental strictness. Respondents were not parents, but rather adolescent and adult children. The parental warmth dimension was measured using the 20 items of the Warmth/Affection Scale (WAS) (Rohner, 2005; Rohner, 1990; Rohner et al., 1978). It assesses the degree to which parents are affectionate, responsive, and involved in their child’s affairs. A sample item for the adolescent group is “Make it easy for me to tell them things that are important to me” while for the young adult group is “Made it easy for me to tell them things that were important to me”. For young adult children, there is an adult version that contains identical statements in the past tense. The Warmth/Affection Scale has a 4-point Likert-type scale ranging from 1 *Almost never is/was true* to 4 *Almost always is/was true*. Higher scores on this scale signify greater parental warmth. The alpha value was 0.941. The parental strictness dimension was measured using the 13 items of the Parental Control Scale (Rohner, 2005; Rohner, 1990; Rohner et al., 1978). It evaluates the degree to which children and adult children perceive that their parents tend to control and monitor their behavior in a strict, imposing, firm, and demanding manner. A sample item for the adolescent group is “Like tell me what to do all the time”, while for the young adult group is “Liked to tell me what to do all the time”. In the same way, for the Parental Control Scale, there is an adult version for young adult children that contains identical statements in the past tense. The Parental Control Scale has a 4-point Likert-type scale ranging from 1 *Almost never is/was true* to 4 *Almost always is/was true*. Higher scores on this scale signify higher parental strictness. The alpha value was 0.887.

Both the Warmth/Affection Scale and the Parental Control Scale have good psychometric properties. They are reliable and valid measures for children to evaluate parental socialization both as it occurs and in the long term, i.e., the extent of warmth and strictness used by their parents during the socialization process and beyond (Villarejo et al., 2024; Martinez-Escudero et al., 2023; Rohner and Khaleque, 2003; Khaleque and Rohner, 2002b; Khaleque and Rohner, 2002a). These scales are widely used in studies around the world (Villarejo et al., 2024; Reyes et al., 2023; Gomez and Rohner, 2011). The Warmth/Affection Scale has been utilized in nearly 500 studies over the past five decades globally. A meta-analysis has been published based on 66 studies reviewing international research, including European countries (Khaleque and Rohner, 2012; Rohner and Khaleque, 2005). The Parental Control Scale has been applied in five culturally diverse contexts (Rohner and Khaleque, 2003), and in several European countries (Reyes et al., 2023; Calafat et al., 2014).

#### 2.2.2 Psychosocial adjustment

Emotional self-concept was assessed using the 6 items from the emotional scale of the AF5 Self-Concept Form 5 (Chen et al., 2020; Garcia and Musitu, 1999). This scale evaluates the broad self-perception of emotional status and the response to specific daily situations that require a certain degree of commitment and involvement (Yepes-Baldó et al., 2025; Fuentes et al., 2020). A sample reverse item is “I get scared easily”. The response scale is a 99-point scale, ranging from 1 *Very little agreement* to 99 *Very much agreement*. Higher scores on this scale are associated with a higher emotional self-concept. The alpha value was 0.763.

Self-esteem was assessed using the 10 items of the Rosenberg questionnaire (Rosenberg, 1965). This instrument assesses the extent of feelings of self-worth, self-respect, and self-acceptance (Villarejo et al., 2024; Martinez-Escudero et al., 2023; Rosenberg, 1965). A sample item is “I take a positive attitude toward myself”. It consists of a 4-point Likert-type response scale ranging from 1 *Strongly disagree* to 4 *Strongly agree*. Higher scores on this scale is associated to greater self-esteem. The alpha value was 0.869.

Social competence was assessed using the 8 items of the social competence scale from the Psychosocial Maturity Questionnaire (CRPM3) (Zacares and Serra, 1996; Greenberger et al., 1975). It evaluates the development of successful interpersonal relationships with peers and adults (Villarejo et al., 2024; Baumrind, 1978; Greenberger et al., 1975). A sample item is “I consider myself affectionate, warm and open in personal relationships”. The scale consists of a 5-point Likert-type response format ranging from 1 *Very inadequate to describe me* to 5 *Very suitable to describe me*. Higher scores on this scale correspond to greater social competence. The alpha value was 0.828.

Conformity was assessed using the 4 items from Schwartz’s Values Inventory (Schwartz and Bilsky, 1987). It assesses the extent to which individuals restrain unruly impulses and inhibit actions that might harm others’ interests (Schwartz and Bilsky, 1987). A sample item is “Respectful (Showing consideration and honor)”. The response scale consists of a Likert-type scale ranging from 1 *Not at all important in my life* to 99 *Essential in my life*. Higher scores on this scale represent greater human values of conformity. The alpha value was 0.717.

### 2.3 Data analysis

The statistical analyses carried out were correlation analysis and multiple linear regression analysis. Correlation analysis was performed between the two main parenting dimensions (i.e., warmth and strictness) and four child adjustment criteria (i.e., emotional self-concept, self-esteem, social competence, and conformity). These correlations were measured separately in the two age groups (i.e., adolescent children and young adult children). A multiple linear regression model was performed for each child adjustment criteria. The predicted variables were the four child adjustment criteria (i.e., emotional self-concept, self-esteem, social competence, and conformity) and the predictors were the two main parenting dimensions (i.e., warmth and strictness), age and sex.

## 3 Results

### 3.1 Relation between parenting and child adjustment

Correlations between parenting and adjustment variables for adolescent and young adult children revealed some statistically significant results (*p* < 0.05; see Table 2). Higher parental warmth was significantly associated with higher social competence (*r* = 0.276, *d* = 0.574; *p* < 0.01) and conformity (*r* = 0.298, *d* = 0.624; *p* < 0.01) in the adolescent sample. Similarly, in the young adult sample, higher parental warmth was significantly associated with higher self-esteem (*r* = 0.196, *d* = 0.400; *p* < 0.01), social competence (*r* = 0.237, *d* = 0.488; *p* < 0.01), and conformity (*r* = 0.210, *d* = 0.430; *p* < 0.01). In contrast, higher parental strictness was significantly associated with lower conformity in the adolescent sample (*r* = −0.125, *d* = 0.252; *p* < 0.01). Higher parental strictness was also significantly associated with lower emotional self-concept in the young adult sample (*r* = −0.135, *d* = −0.272; *p* < 0.01).

**Table 2:** Correlations between parental dimensions and child adjustment #.

	1	2	3	4	5	6
1. Parental warmth		−0.266^** ϯϯ^	0.047	0.051	0.276^** ϯϯ^	0.298^** ϯϯ^
2. Parental strictness	−0.182^** ϯ^		−0.077	−0.025	−0.008	−0.125^** ϯ^
3. Emotional self-concept	0.043	−0.135^** ϯ^		0.283^** ϯϯ^	0.221^** ϯ^	−0.078
4. Self-esteem	0.196^** ϯ^	−0.076	0.291^** ϯϯ^		0.188^** ϯ^	0.004
5. Social competence	0.237^** ϯ^	−0.009	0.233^** ϯ^	0.270^** ϯϯ^		0.225^** ϯ^
6. Conformity	0.210^** ϯ^	0.003	−0.022	0.080	0.196^** ϯ^	

^# Correlations for adolescents are shown above the diagonal and for young adults below the diagonal.^

*p < 0.05; **p < 0.01.

^ϯ^ d (0.20, 0.49) small effect size; ^ϯϯ^ d (0.50, 0.79) medium effect size.

Additionally, adolescents with higher emotional self-concept reported higher self-esteem (*r* = 0.283, *d* = 0.590; *p* < 0.01) and social competence (*r* = 0.221, *d* = 0.453; *p* < 0.01). Higher social competence was significantly associated with higher self-esteem (*r* = 0.188, *d* = 0.383; *p* < 0.01) and conformity (*r* = 0.225, *d* = 0.462; *p* < 0.01) in the adolescent sample. Similarly, young adults with higher emotional self-concept showed higher self-esteem (*r* = 0.291, *d* = 0.608; *p* < 0.01) and social competence (*r* = 0.233, *d* = 0.479; *p* < 0.01). Higher social competence was significantly associated with higher self-esteem (*r* = 0.270, *d* = 0.561; *p* < 0.01) and conformity (*r* = 0.196, *d* = 0.400; *p* < 0.01) in the young adult sample.

### 3.2 Predictions of child adjustment depending on parenting, age, and sex

**Figure 1:**
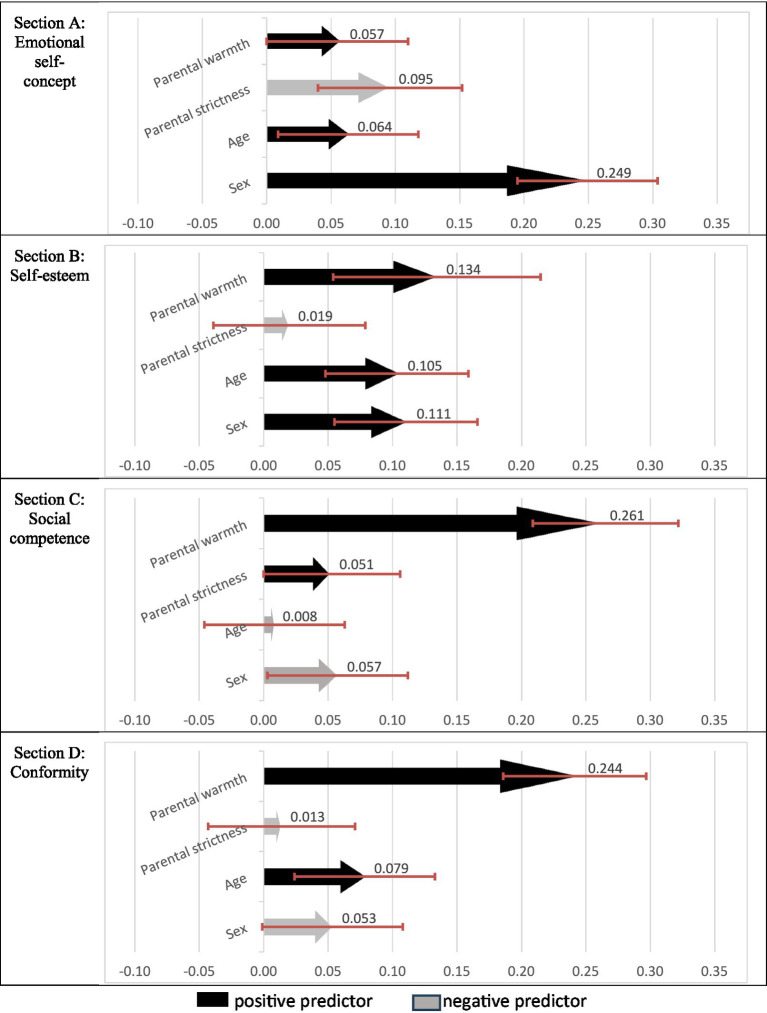
95% confidence intervals for regression beta values. The sign and order of the lower and upper limits of the beta confidence intervals of the negative predictors have been reversed so that the arrows of all predictors go in the same direction and the differences in the magnitude of each predictor can be clearly seen.

All four predictive models of the adjustment components were statistically significant (*p <* 0.001; see Table 3). Specifically, multiple linear regression models were performed for each child adjustment component (i.e., emotional self-concept, self-esteem, social competence, and conformity) depending on parenting dimensions (i.e., warmth and strictness), age, and sex. Results revealed differences between predictors on child adjustment components as well as variations in magnitude based on effect size (see Figure 1).

**Table 3:** Multiple linear regression coefficients between parenting dimensions, sex and age, and child adjustment.

Dependent variable	Predictors	*b* (95%)	*SE*	Βeta (95%)	*t*	*d*
Emotional self-concept *R*^2^_adj_ = 0.072 *F*(4, 1,219) = 24.85^***^	Warmth	0.009 (0.001, 0.018)	0.005	0.057 (0.001, 0.110)	1.99^*^	0.114
	Strictness	−0.021 (−0.034, −0.009)	0.006	−0.095 (−0.152, −0.040)	−3.32^***^	0.190
	Age	0.222 (0.033, 0.411)	0.097	0.064 (0.009, 0.118)	2.30^*^	0.132
	Sex	0.881 (0.688, 10.074)	0.098	0.249 (0.195, 0.304)	8.97^***^	0.514^ϯϯ^
Self-esteem *R*^2^_adj_ = 0.036 *F*(4, 1,219) = 12.42^***^	Warmth	0.002 (0.001, 0.004)	0.001	0.134 (0.054, 0.215)	4.62^***^	0.265^ϯ^
	Strictness	0.000 (−0.002, 0.001)	0.001	−0.019 (−0.079, 0.039)	−0.66	0.038
	Age	0.041 (0.019, 0.063)	0.011	0.105 (0.048, 0.159)	3.70^***^	0.212^ϯ^
	Sex	0.045 (0.022, 0.067)	0.011	0.111 (0.055, 0.166)	3.92^***^	0.225^ϯ^
Social competence *R*^2^_adj_ = 0.069 *F*(4, 1,219) = 23.52^***^	Warmth	0.016 (0.013, 0.020)	0.002	0.261 (0.209, 0.322)	9.11^***^	0.522^ϯϯ^
	Strictness	0.004 (0.001, 0.009)	0.002	0.051 (0.000, 0.106)	1.80	0.103
	Age	−0.011 (−0.083, 0.061)	0.037	−0.008 (−0.063, 0.046)	−0.29	0.017
	Sex	−0.077 (−0.150, −0.004)	0.037	−0.057 (−0.112, −0.003)	−2.06^*^	0.118
Conformity *R*^2^_adj_ = 0.071 *F*(4, 1,219) = 24.37^***^	Warmth	0.033 (0.025, 0.040)	0.004	0.244 (0.186, 0.297)	8.54^***^	0.489^ϯ^
	Strictness	−0.002 (−0.013, 0.008)	0.005	−0.013 (−0.071, 0.043)	−0.44	0.025
	Age	0.225 (0.069, 0.382)	0.080	0.079 (0.024, 0.133)	2.83^**^	0.162
	Sex	−0.156 (−0.315, 0.003)	0.081	−0.053 (−0.108, 0.001)	−1.92	0.110

^*^p < 0.05; ^**^p < 0.01; ^***^p < 0.001.

^ϯ^d (0.20, 0.49) small effect size; ^ϯϯ^d (0.50, 0.79) medium effect size.

The first predictive model was for emotional self-concept. The two parenting dimensions were statistically significant predictors but in different directions. Warmth was a positive predictor (β = 0.057, *d =* 0.114; *p < 0*.05), indicating that higher parental warmth predicted higher emotional self-concept. Conversely, parental strictness was a negative predictor (β = −0.095, *d =* 0.190; *p < 0*.001), revealing that increased parental strictness predicted lower emotional self-concept. Age was a significant positive predictor (β = 0.064, *d =* 0.132; *p <* 0.05); young adults showed higher emotional self-concept than adolescents. Sex was also a significant positive predictor (β = 0.249, *d =* 0.514; *p <* 0.001). Males had higher emotional self-concept than females. The contribution of sex to emotional self-concept was the largest among the predictors, as there was no overlap of its intervals with those of the other three predictors (see Figure 1, section A).

The second predictive model concerned self-esteem. Parental warmth was a positive predictor of self-esteem (β = 0.134, *d =* 0.265; *p < 0*.001), indicating that greater parental warmth predicted higher self-esteem. Parental strictness had no significant contribution to self-esteem (β = −0.019, *d =* 0.038; *p >* 0.05). Regarding age and sex, age was a significant positive predictor (β = 0.105, *d =* 0.212; *p <* 0.001), indicating that young adults had higher scores than adolescents. Similarly, sex was a significant predictor (β = 0.111, *d =* 0.225; *p <* 0.001), indicating that men showed higher self-esteem than women. The greatest contribution of the predictors to self-esteem was identified in parental warmth, although it did not differ in magnitude with the other two predictors (age and sex) because the confidence intervals overlapped (see Figure 1, section B).

The third predictive model was for social competence. Among parental predictors, only warmth reached statistical significance (β = 0.261, *d =* 0.522; *p <* 0.001), while strictness did not (β = 0.051, *d =* 0.103; *p >* 0.05). The greater the parental warmth, the greater the social competence. Sex (β = −0.057, *d =* 0.118; *p <* 0.05), but not age (β = −0.008, *d =* 0.017; *p >* 0.05), was a significant predictor, with females reporting greater social competence than males. Parental warmth showed the greatest contribution to social competence among the statistically significant predictors, as the confidence interval for parental warmth did not overlap with those of the others (see Figure 1, section C).

The fourth predictive model was for conformity. Again, parental warmth was a statistically significant predictor (β = 0.244, *d =* 0.489; *p <* 0.001), indicating that the greater the warmth, the greater the conformity. In contrast, parental strictness had no significant contribution to conformity (β = −0.013, *d =* 0.025; *p >* 0.05). Age was a significant positive predictor (β = 0.079, *d =* 0.162; *p <* 0.01), but sex was not (β = −0.053, *d =* 0.110; *p >* 0.05); young adults showed greater conformity than adolescents. However, parental warmth, as in social competence, had the most significant contribution to conformity, with the confidence interval for warmth not overlapping with that for age (see Figure 1, section D).

## 4 Discussion

Based on the two-dimensional theoretical framework (Maccoby and Martin, 1983), the present study examines among adolescent and young adult children the relationship between the two main parenting dimensions (i.e., warmth and strictness) and child psychosocial adjustment (captured by emotional self-concept, self-esteem, social competence and conformity). According to the present findings, parental warmth contributed positively to the self-perceptions about emotional regulation (emotional self-concept) and personal worth (self-esteem), as well as social competence and conformity values. Parental strictness contributes neither to social competence and conformity to social norms nor to self-perceptions (in terms of self-esteem) and may even severely harm the emotional component (i.e., emotional self-concept). This pattern was relatively similar for adolescent children as for young adult children.

It is conjectured that the influence of the first authority figure (i.e., parents), which can be either positive or negative, is crucial in conformity to social norms (Koerner and Fitzpatrick, 2002; Baumrind, 1978). Parents can act as either protective or risk factors in transmitting social values to their children, helping them become competent adults who are psychosocially adjusted to the cultural context in which they live (Grusec and Goodnow, 1994). The present study clearly identifies the relationship between parental warmth and strictness with emotional self-concept, self-esteem, social competence, and conformity values. Conformity values represent a parenting outcome that has been less studied, as previous research has typically focused on the practices that parents employ to encourage conformity to family norms without measuring whether children have internalized norms that represent self-restraint in their own behavior to respect others (Schrodt et al., 2007; Koerner and Fitzpatrick, 1997). In this sense, family goes beyond merely transmitting certain norms for proper home functioning; rather, the family instills particular social values that allow children to conform to social norms and reject deviant standards: the values of conformity. The priority children give to restraining their actions, tendencies, and impulses to avoid disrupting or harming others (i.e., conformity values) is necessary for an adequate functioning in society, enabling individuals to relate correctly with others in any context (e.g., peers, school), including those in which parents cannot be present (Katz-Gerro et al., 2017; Oarga et al., 2015; Christensen et al., 2004). Interestingly, for both adolescent and adult children, protective and risk parenting factors related to conformity values are in line with those of the other components of psychosocial adjustment studied: one social (social competence) and two related to the self (emotional self-concept and self-esteem).

As theories of socialization point out, parental presence cannot be constant and ubiquitous; instead, parents must prepare their children to live in society (Rudy and Grusec, 2001; Bugental and Goodnow, 1998; Darling and Steinberg, 1993; Maccoby, 1992; Baumrind, 1978). A parent–child relationship characterized by affection, support, and trust—rather than psychological control and surveillance—can provide children with the security and confidence they need to face difficulties and obstacles. Family trust helps children develop self-confidence and conform to social norms, enabling them to manage situations effectively, especially when monitors cannot be present. Consequently, children can internalize social values and interact positively with their peers, avoiding disruptive behaviors within the peer group. Parental socialization is particularly important during adolescence, as adolescent children spend significant amounts of time away from their parents but in the company of their peers. But parenting years are equally crucial in adulthood, where the roots of their previous upbringing will partially explain their success or failure in the adult world. According to the findings of the present study, which analyzed child adjustment by age, adolescents have more difficulties than young adults in terms of lower emotional self-concept, self-esteem, and conformity values, thereby confirming some previous research (Alcaide et al., 2023; Vecchione et al., 2020; Klein, 1972). However, as the present study revealed, adolescent children who reported more positive scores are those raised in homes characterized by warmth rather than parental strictness, a pattern that was also observed in young adult children, once parental socialization is over.

According to previous parenting studies, mainly from middle-class European-American families, parental warmth would be benefit for child development, but only accompanied with strictness (Darling and Steinberg, 1993; Lamborn et al., 1991). However, the present study seriously questions the findings on the benefits of parental strictness for improving individuality within social norms, without showing problems and maladjustment (Lamborn et al., 1991; Patterson and Stouthamerloeber, 1984). Social competence and conformity values are both protective factors against deviant behaviors (Bilsky et al., 2011; Schwartz, 2006; Holder, 1958). Although previous classic European-American studies indicate a positive relationship between parental strictness and social competence and conformity values, the current study conducted in Spain showed that parental strictness does not significantly contribute to these areas; instead, parental warmth alone was a protective factor. These results align with more recent research primarily conducted in European and Latin-American countries, identifying parental warmth—without the accompaniment of strictness—as a protective factor against problems such as alcohol (Garcia et al., 2020b), drug use (Villarejo et al., 2024; Riquelme et al., 2018), and even delinquency (Climent-Galarza et al., 2022).

The present results also provide additional evidence regarding the relationship between parenting and emotional regulation. Parental warmth—supported by evidence from European-American studies—has been suggested to benefit emotional regulation, which is common in both indulgent and authoritative parenting styles. However, surprisingly, adolescents from indulgent homes have scored negatively on various emotional outcomes, likely due to issues such as drug use related to a lack of strictness (Steinberg et al., 1994; Lamborn et al., 1991). According to the present findings, parental strictness, a key component of the authoritative style, is associated with emotional regulation problems, particularly in relation to emotional self-concept (higher parental strictness is associated with lower emotional self-concept). Emotional self-concept acts as a protective factor against mental health and well-being problems (Sticca et al., 2020). Interestingly, this study identifies that to help children develop the self-confidence needed to cope with stressful situations in daily life (i.e., a high emotional self-concept), parents must be affectionate and nurturing (i.e., showing high parental warmth), while avoiding high levels of control and surveillance (i.e., showing low parental strictness). In terms of self-esteem, parental strictness does not contribute significantly; rather, only parental warmth predicts higher self-esteem scores. Moreover, this study suggests that high parental strictness may actually undermine the benefits of high warmth, acting as a risk factor for emotional self-concept and increasing the child’s vulnerability to mental health and well-being issues (Sticca et al., 2020).

The present study agrees with the literature indicating that the so-called positive parenting (the authoritative style, defined by high parental strictness and high parental warmth) may not be beneficial in all contexts (Palacios et al., 2022; Garcia et al., 2019; Garcia and Gracia, 2009; Chao, 2001; Darling and Steinberg, 1993). Previous cross-cultural research points out that the same parenting style (e.g., authoritative and authoritarian) might have different consequences depending on the culture in which the family lives (Martinez et al., 2021; Deater-Deckard et al., 1996; Baumrind, 1972). As observed in the present study, recent empirical evidence, primarily from Europe and Latin American countries, suggests that parental warmth serves as a protective factor for child adjustment (Martinez-Escudero et al., 2023; Climent-Galarza et al., 2022), while parental strictness does not contribute positively and may even undermine the benefits of warmth in protecting against deviance and maladjustment (Villarejo et al., 2024; Alcaide et al., 2023). Parental warmth could be as effective or even more so than parental strictness, which appears unnecessary for children’s healthy development. Warmth acts as a protective factor for academic adjustment (Villarejo et al., 2024) or self-perceptions (Fuentes et al., 2022). Even in cultures such as China where parental strictness, even unnacompanied by parental warmth, was traditionally considered to be related to optimal child adjustment, current studies in that culture show that parental strictness alone is related to poor levels of child psychosocial adjustment (Chao, 2001; Ho, 1986). In contrast, these current studies in the Chinese culture show that parental warmth is the dimension related to optimal child adjustment, alone or accompanied by parental strictness (Chen et al., 2024). Parenting styles and their effects on child adjustment may differ across cultural contexts (Pinquart and Lauk, 2024; Pinquart and Kauser, 2018). The use of parental strictness, although recommended for children from European-American families, may be ineffective or even harmful in European and Latin American countries, even when accompanied by warmth. This may be partly due to the more egalitarian nature of parent–child relationships, where individuals are more interdependent with the groups to which they belong (e.g., family, nation) and prioritize group goals over their own (i.e., horizontal collectivistic cultures). It is argued that, for this reason, parental strictness may be perceived by children in European and Latin American countries as a deprivation of their freedom, leading to a lack of association between strictness and positive child adjustment (Garcia et al., 2021; Calafat et al., 2014).

Parental warmth creates an environment of acceptance and fosters two-way communication, providing children with the security and confidence to disclose personal information. This approach allows parents to understand different aspects of their children’s lives without resorting to intrusive monitoring or imposition. High levels of parental knowledge about their children’s lives have been associated with several indicators of positive child adjustment. Research suggests that spontaneous disclosures from children may contribute more significantly to these positive outcomes than parental monitoring with surveillance efforts (Kerr and Stattin, 2000). Evidence indicates that effective parent–child communication is more beneficial than surveillance and psychological control (Stattin and Kerr, 2000). It is possible that when parents promote open communication, develop cordial relationships, show genuine interest in their children’s problems, and provide reasoned explanations for the consequences of their actions (through parental warmth), they help children understand and internalize social norms, thereby enabling them to become mature and responsible adults (Garcia et al., 2020b). Furthermore, the reasoning and openness inherent in parental warmth allow children to feel loved and valued within the family (Ertema et al., 2025; Martinez et al., 2021). A child’s perception of being loved and valued is shaped by their interpretation of family experiences and is closely related to parental behaviors and attitudes, particularly warmth (Krauss et al., 2020). Several studies have indicated that this perception is positively associated with various aspects of child adjustment, including lower levels of emotional instability, antisocial behavior, psychological distress, and depressive symptoms, as well as higher academic performance (Ertema et al., 2025; Martinez et al., 2021).

The present study has important strengths, but some limitations should be considered. Due to the long duration of time that has elapsed between parental socialization (when the child has been raised by their parents) and the moment when the child (i.e., offspring) is a young adult, caution is warranted since this study is based on cross-sectional data rather than longitudinal data. However, some longitudinal studies on parental socialization show similar results, parental warmth during the years of parental socialization is positively related to the child’s psychosocial adjustment in adulthood (Hintsanen et al., 2019; Eisenberg et al., 2015). Additionally, other classic and recent studies on parenting have used cross-sectional rather than longitudinal designs and have established relationships between parental socialization and child adjustment in both the short term (i.e., adolescence) and the long term (i.e., adulthood). These cross-sectional studies also show results consistent with the present study, showing that parental warmth is positively related to child adjustment, while parental strictness tends to be unnecessary or even detrimental (Koutra et al., 2022; Meldrum et al., 2015). Additionally, it is worth mentioning that no experimental methodology has been used in this study. Furthermore, the age ranges for adolescents and young adults differ significantly, with the adolescent age range being 6 years compared to 16 years for young adults, reflecting the shorter duration of adolescence in contrast to young adulthood. Other family studies have focused on even shorter durations within emerging adulthood (Padilla-Walker et al., 2021; Rote et al., 2020). Although longitudinal studies are more common in adolescents, future studies should continue the follow-up throughout young adulthood, where the evidence is much more limited (Aquilino and Supple, 2001).

The present findings confirm the impact (positive or negative) of the family even beyond adolescence, when the child is a young adult and has to face difficult challenges such as career development or starting a family. Other studies have also identified the influence of parental socialization in the long term, i.e., on the adjustment of adult children, but have not done so by comparing the adjustment of adolescent and adult children at the same time. When the parental socialization process has been completed and the child is an adult, the parents may have had a positive (protective factor) or negative (risk factor) impact on this child’s adjustment. This study is conducted based on four adjustment indicators that can be evaluated throughout different stages of life, such as adolescence or adulthood, and also include aspects of the self (self-esteem, emotional self-concept) and the relationship with others (social competence and conformity). Besides, the respondents of the questions about child adjustment and parenting dimensions were the children because children seem to offer more reliable and accurate data than other sources such as parents (Barry et al., 2008; Gonzales et al., 1996). The present study analyzes, in addition to the existence of a positive or negative relationship between parenting dimensions and child adjustment criteria, the effect size of this relationship. What is most interesting is that, in general, adjusted children (adolescents and young adults) have benefited from parental warmth during the years of parental socialization, not from parental strictness.

## Data Availability

The original contributions presented in the study are included in the article/supplementary material, further inquiries can be directed to the corresponding author.
